# Age and Fertility: A Study on Patient Awareness

**DOI:** 10.5935/1518-0557.20160024

**Published:** 2016

**Authors:** Sara Deatsman, Terrie Vasilopoulos, Alice Rhoton-Vlasak

**Affiliations:** 1 Department of Obstetrics and Gynecology. Division of Reproductive Endocrinology and Infertility. University of Florida; 2 Department of Anesthesiology, University of Florida, Gainesville, Florida

**Keywords:** Infertility, Advanced maternal age, Fertility

## Abstract

**Objective:**

Fertility declines as women age. Advancing maternal age increases pregnancy risks such as diabetes or hypertension. Studies suggest women are not aware of the risks of aging on fertility and pregnancy. The study objective was to assess women's knowledge of fertility and reproductive outcomes affected by aging.

**Methods:**

Prospective IRB approved survey of women (n=94) attending an obstetrics and gynecology (OB/GYN) clinic. Data collected included demographics, pregnancy history, and knowledge of age-related fertility decline and pregnancy risks. Statistical analysis performed using JMP Pro11.0.

**Results:**

Ages ranged from 18 to 67. One third (30.5%) were aware fertility begins to decline at age 35, however this varied among groups depending on prior history of infertility or requiring fertility treatment. Nulliparous women were more unaware of the health risks of pregnancy over age 35 (1.4% vs 13.6%, *P* 0.02). African Americans (AA) women were less likely to think obesity (76% Caucasian vs 47.8% AA vs 66.7% other, *P* < 0.05) and older age (88% Caucasian vs 60.9% AA vs 82.7% other, *P* 0.02) affected fertility.

**Conclusion:**

Knowledge regarding fertility and reproduction related to aging was variable and differed by age and race. Difficulty conceiving appears to be associated with higher knowledge levels. Public education will increase awareness of age-related fertility declines. Increased contact during pregnancy is an excellent opportunity to educate women in a nondirective way.

## INTRODUCTION

Fecundity in females begins to decrease during the 4th decade of life,(ACOG, 2014) with subfertility becoming more pronounced at age 35(Maheshwari *et al*., 2008). Fertility declines with increasing maternal, as early as 32, and especially after the mid-30's. For this reason delayed child-bearing is traditionally defined as pregnancy occurring in women over 35 years of age (Johnson & Tough, 2012). Fecundity decreases primarily due to oocyte atresia and is compromised prior to the onset of peri-menopausal menstrual irregularities. The best surrogate marker for oocyte quality is age, (ACOG, 2015) though patients often feel that health and fitness are better indicators of fertility (Daniluk *et al*., 2012). The decline in fertility is accompanied by an increased risk of aneuploidy and spontaneous abortions (ACOG, 2014). Pregnancies in older women have increased risk of other issues including gestational diabetes, hypertensive disorders, placenta previa, operative delivery and maternal mortality (Maheshwari *et al*., 2008). A review of current literature reveals that some women are aware of age-related effects on pregnancy (Maheshwari *et al*., 2008), while others are unaware there is an effect (Lundsberg *et al*., 2014).

The National Vital Statistics birth data illustrate a continued upward trend in delayed childbearing. The birth rate for women aged 35-39 was 49.3 births per 1,000 women in 2013, up 2% from 2012. The 2013 rate is the highest since 1964. The birth rate for women aged 40 - 44 was 10.4 births per 1,000 women in 2013. The rate for women 40 - 44 generally has risen over the past three decades by more than 400%. The birth rate for women aged 45-49 (which includes births to women aged 50 and over) was 0.8 births per 1,000 in 2013, which is a 14% rise in one year (Martin *et al*., 2015). Delayed childbearing has increased the proportion of women seeking to have their first births at older ages when infertility is more likely, thus making this information even more important given these societal birth change trends.

The American College of Obstetrics and Gynecology (ACOG) and American Society for Reproductive Medicine (ASRM) encourage counseling reproductive age women about age-related fertility decline and pregnancy risks and provides timelines for evaluation for infertility (ACOG, 2014). They also recommend that clinicians should encourage women to consider their pregnancy plan at each visit. The Center for Disease Control and Prevention (CDC) encourages women to develop a "Reproductive Life Plan" that they discuss with their healthcare provider (CDC, 2014). Per current literature, patients prefer to receive this information from their health care providers or from a web based program (Daniluk *et al*., 2012). The purpose of this study was to assess the knowledge level and awareness of the effects of reproductive aging in a cohort of women attending a general OB/GYN clinic. We hypothesized that women having greater exposure to healthcare providers, such as participants who have been pregnant, might have greater awareness due to more healthcare visits for counseling and health education.

## MATERIALS AND METHODS

### Subjects

The study was an IRB approved anonymous 17-question non-validated survey (attached as supplementary material). An informed consent was included. All new patients over 18 years of age presenting for obstetric or non-oncologic gynecology visits between October 2014 and March 2015 were eligible for participation. Survey participation was offered to women at 2 clinical sites at the time of check in for their visits. The surveys were completed once the patients had been roomed in clinic and then placed in a marked box at the checkout desk. There were no other inclusion or exclusion criteria.

The reproductive health survey was developed for the purpose of this study and contained questions pertaining to the following: demographic information (2 items), prior pregnancy and infertility history (6 items), knowledge of factors including age that reduce fertility (5 items), risks of advanced maternal age (1 item), whether they had a pregnancy plan (2 items), and preferred source for health related fertility information (1 item). The question types included fill in the blank, yes/no questions, and choose from a list of options. The survey required about 5 minutes to complete. While not validated, the internal consistency of the survey questions was high (Cronbach's alpha=xxx).

### Statistics

Summary and comparison statistics were performed using JMP Pro 11.0 (SAS Institute, Cary, NC). Chi square tests were used to compare differences patient's knowledge on issues affecting fertility (i.e. frequencies of response to survey questions) across demographic variables (age group, race, history of prior pregnancy, and history of difficulty conceiving, history of prior fertility treatment). A *P* of <.05 was considered statistically significant.

## RESULTS

Ninety seven surveys were collected; one survey was excluded due to patient age <18 years and 2 for incomplete answers. Demographic information is summarized in Table 1. The participants ranged in age from 18 to 67 years with the mean age of the participants being 30.9 ± 9.2 years. The majority of participants were Caucasian (54.3%). A majority (75.8%) of women reported a history of prior pregnancy. Nearly twenty percent reported difficulty conceiving, with 8.5% reporting that they required fertility treatment. One third of the women (30.5%) stated that fertility begins to decline at age 35; however 5.3% stated they believed there was no age where fertility declines.

**Table 1:** Participants Demographics

Demographics of sample	N=94
	Mean (SD) or %
Age (years)	30.9 (9.2)
Race/Ethnicity (%)	
Caucasian (non-Hispanic)	54.3%
African American	23.5%
Asian	4.3%
Native American	1.1%
Do not wish to disclose	3.2%
Hispanic	11.7%
Have ever been pregnant (% yes)	75.8%
Live births (% any)	86.8%
Miscarriages (% any)	16.7%
Have living children now (% yes)	65.3%
Plan to have children (% yes)	68.1%
Had difficulty getting pregnant(% yes)	18.1%
Required fertility treatment (% yes)	8.5%

Most participants endorsed knowledge of different factors that could reduce fertility including smoking (68.4%), obesity (69.5%), alcohol use (67.4%), older age (78.95%), and history of STI (72.6%). The majority also endorsed knowledge of health risks in pregnancy over age 35 including diabetes (69.1%), miscarriage (79.8%), high blood pressure (75.56%), and genetic abnormalities (74.5%), however 4.3% reported no increased health risks in pregnancy over age 35.

Only 35.6% of the participants reported having a pregnancy plan in place; 48.3% reported they would change their plan if they knew age affected fertility. Most participants were getting their fertility information from their doctor (34%) or a friend (31.9%). They preferred websites (54.3%), brochures (39.3%), and discussions with providers (38.3%) as a way to receive fertility information.

Differences in fertility knowledge were then evaluated looking at different categories: history of prior pregnancy, history of prior difficulty conceiving, history of prior fertility treatment, age and race.

**Figure 1:**
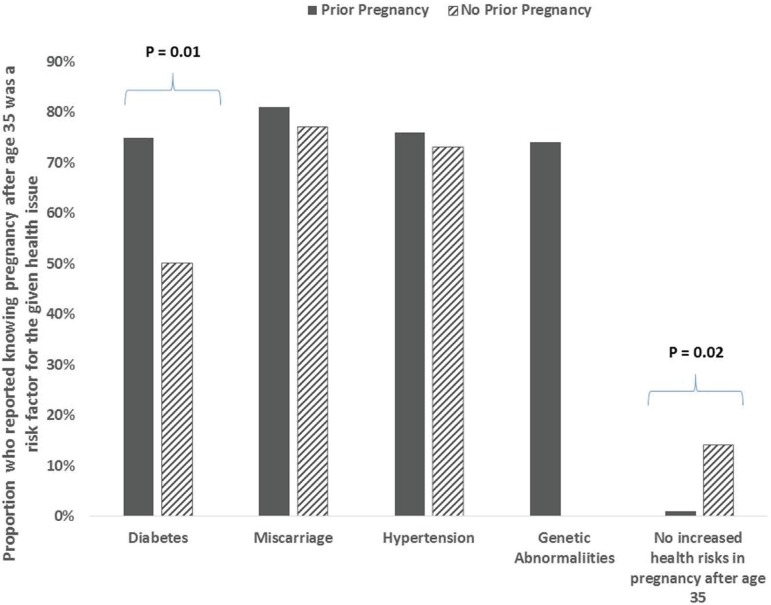
Awareness of Health Risks in Pregnancy over Age 35. Proportion who reported knowing pregnancy after 35 was a risk factor for the given health issue.

As mentioned above, 75.8% of the study population reported a history of prior pregnancy, 86.8% had live births and 16.7% had miscarriages. When the prior pregnancy group was compared to those who had never been pregnant, there was no significant difference when asked about age of fertility decline. Both groups agreed that a number of factors could lead to infertility (smoking, obesity, alcohol use, older age). Patients with a history of pregnancy were more likely to say that history of an STI could lead to fertility issues (79.2% vs 52.2%, *P* 0.01) and that diabetes risk was increased in pregnancy over age 35 (75% vs 50%, *P* 0.03). The group that had not been pregnant was significantly more likely to say there are no increased risks in pregnancy over age 35 (13.6% vs 1.4%, *P* = 0.02) (Figure 1).

Nearly 20 percent of the study group reported difficulty conceiving; 50.0% of these women underwent fertility treatment; 80.0% had live births and 36.4% had miscarriages. When this group was compared to those who did not have difficulty conceiving, the only significant differences found was age at which fertility declines. Those with a history of difficulty reported fertility declining prior to age 30 (23.5% vs 4.7%, *P* 0.03; 43.8% vs 17.7%, *P* 0.04) or over age 35, while the group without difficulty reported fertility decline at age 30 (35.3% vs 14.1%, *P*=0.04).

8.5% of the participants reported that they required fertility treatments. When they were compared to the rest of the group, they actually were more likely to say that fertility begins to decline prior to age 30 (50% vs 15.9%, *P* 0.02). When asked about increased health risks in pregnancy over the age of 35, 100% of the group who required fertility treatment endorsed diabetes, miscarriage, hypertension and genetic abnormalities as risks. When compared to the rest of the study group, they were more likely to report an increased risk of diabetes (100% vs 69.8%, *P*=0.02), hypertension (100% vs 73%, *P*=0.03), and genetic abnormalities (100% vs 73%, *P*=0.03) in pregnancy over age 35.

To compare knowledge based on age, the group was divided in to under 30 and 30 and over. There were two main differences between these groups. First, the under 30 group was more likely to report that fertility begins to decline in the mid to late thirties compared to the 30 and over group (45.8%vs 24.4%, *P* 0.03). When asked about factors that could affect fertility, there were no significant differences between the groups except those under 30 were more likely to report alcohol use as a factor (83.3% vs 51.1%, *P*=0.0008).

**Figure 2:**
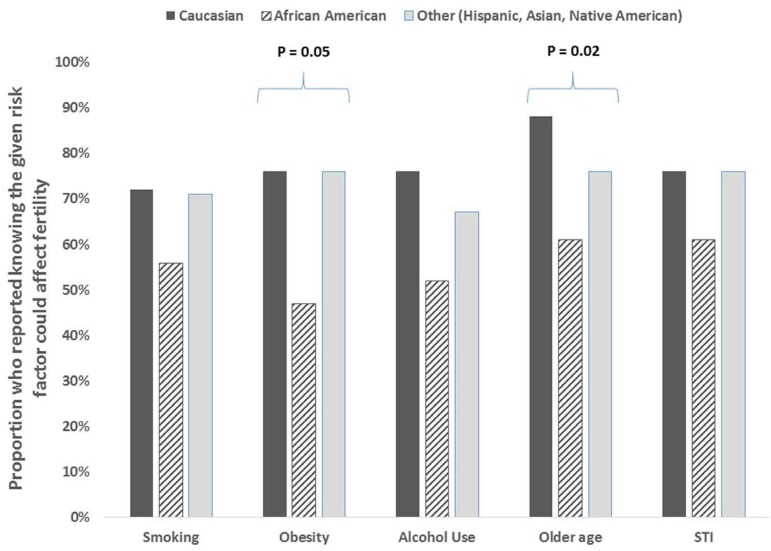
Racial Differences in Awareness of Factors that Could Affect Fertility. Proportion who reported knowing the given risk factor could affect fertility.

**Figure 3:**
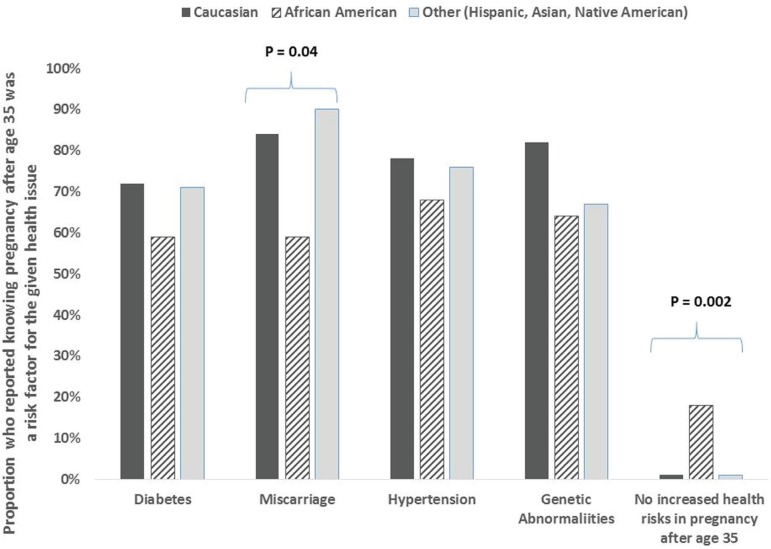
Racial Differences in Awareness of Health Risks in Pregnancy over Age 35. Proportion who reported knowing pregnancy after age 35 was a risk factor for the given health issue.

The study group was divided by race and resulted in three groups: Caucasian, African American (AA), and Asian/Hispanic/Native American/Other. The AA and Other group were much more likely to report fertility decline after the age of 35 when compared to the Caucasian group (39.1% vs 53.3% vs 12%, *P* 0.002). Those who identified as AA were less likely to report age (88% vs 60.9% vs 82.7%, *P* 0.02) and obesity (76% vs 47.8% vs 66.7%, *P* 0.05) as factors affecting infertility. (Figure 2) They were also less likely to report miscarriage as an increased risk in pregnancy over 35 (84.0% vs 59.1% vs 86.7%, *P* 0.04) and much more likely to report no increased risks in pregnancy over age 35 (0% vs 18.2% vs 0%, *P*=0.002) (Figure 3). Interestingly, no African-Americans in our study (0%) reported histories of difficulty conceiving and fertility treatment (Table 2).

**Table2:** Differences in Patient Demographics across Racial and Ethnic Groups

Demographics of sample	Caucasian	African American	Native American or Hispanic	*P* value
	%	%	%	
Age				0.54
Under 30 years	46.9%	52.2%	61.9%	
30 years or older	53.1%	47.8%	38.1%	
Have ever been pregnant (% yes)	80.0%	73.9%	71.4%	0.70
Have living children now (% yes)	68.0%	73.9%	52.4%	0.30
Plan to have children (% yes)	69.2%	55.6%	78.6%	0.38
Had difficulty getting pregnant (% yes)	25.0%	0.0%	27.8%	0.01
Required fertility treatment (% yes)	15.4%	0.0%	7.1%	0.09

Note: *P*-value from Chi-square test

## DISCUSSION

The goal of our study was to assess the awareness of aging on fertility and reproductive outcomes among a group of women attending a general obstetrics and gynecology clinic. We found that patients who had been pregnant tended to have a better understanding of health risks of pregnancy over age 35 versus patients who had never been pregnant, but both groups had the lower knowledge levels of age-related fertility decline. Our findings may be explained by the numerous encounters with the health system during the pregnancy in which pregnancy-related, and not fertility related topics were reviewed. The only difference seen in the group that reported difficulty getting pregnant is that they thought fertility began to decline at a much earlier age than the rest of the group, possibly reflecting the true decline that begins at age 32 rather than 35 (Johnson & Tough, 2012). This was also true for the group that required fertility treatment. This was expected as these patients were assumed to have more contact with the health care system, which should lead to better knowledge about age-related fertility decline. It was surprising that the women who had children, previous difficulty conceiving, or infertility therapy were aware aging impacts on reproduction and reported this occurred at less than 30, but were more aware of increased pregnancy complications over age 35. Maybe health care providers are getting part of the reproductive aging message across but only as it relates to pregnancy outcomes.

The African American participant's results were somewhat unexpected in that they were less likely to say age or obesity affects fertility when compared with other ethnicities including Hispanic and Caucasian. The AA group also had significantly less knowledge of pregnancy declines over 35 and the higher rates of miscarriages. This shows a very specific area where more education is needed about both age related fertility declines and risks of advanced maternal age and obesity on pregnancy.

Our study is unique in that it compared women at all different reproductive life stages, along with women of different ethnicity and parity. Strength of the present study is the inclusion of a diverse demographic group, which is more generally representative of US females of reproductive age. Also, the survey was handed out at 2 facilities - one resident clinic with a large population of Medicaid patients and a faculty clinic where patients usually have private insurance. It would be interesting to see the role education level and level of income may have had in relation to fertility knowledge, although we did not assess this in the current study.

We found, like Lundsberg *et al*. (2014) that there continues to be knowledge gaps and misconceptions surrounding reproductive health and conception. Their study showed that about 1/5 of women were unaware of the effect of increasing age on conception and reproductive outcomes compared to 30% of women being aware of the fertility decline at age 35. 5.3% of our respondents felt that age had no impact on fertility but interestingly when asked if they would change their plan for future pregnancies 48% would change their reproductive plan if they knew fertility was affected so significantly by age. Previous studies among reproductive age respondents, have demonstrated a lack of awareness regarding the age of fertility decline and the probability of conception across selected ages (Lampic *et al.*, 2006; Bretherick *et al*., 2010; Hashiloni-Dolev *et al*., 2011; Peterson *et al*., 2012), In an infertile population over 40 who required In Vitro Fertilization (IVF), 23% indicated that with more information about declining fertility, they might have attempted conception at an earlier age (Mac Dougall *et al*., 2012).

Medical professionals and society in general should support women achieving their individual reproductive goals, whether those goals include a desire for many children or none at all. Healthcare providers have a critical mission to widely disperse information about age-related fertility decline, as more women delay childbearing to pursue careers, achieve financial security, or await the presence of an appropriate partner. National Vital Statistics birth data confirm the upward trend of delayed childbearing (Martin *et al*., 2015). Reproductive aged women need to have the appropriate knowledge about age-related fertility decline and the inability of reproductive technologies to overcome diminished fecundity. Unfortunately, women often erroneously believe that IVF can reverse the effects of age-related fertility decline (Maheshwari *et al*., 2008).

The American Society for Reproductive Medicine and the American College of Obstetricians and Gynecologists have made recommendations including improved education and enhanced awareness of the effects of age on fertility, both of which are essential in counseling women who desires pregnancy (ACOG, 2014). In order to optimize this information transfer to women, providers need to better incorporate reproductive based discussions at health care visits.

This topic is highlighted in an editorial titled "The importance of 'the fertility talk' " for OBGYNs, family practitioners, or internal medicine physicians. This is a valuable discussion to initiate when female patients are seen in their reproductive years, but it is important to make it a spontaneous and non-judgmental conversation. The discussion highlights the often touchy and delicate nature of conversations about reproductive aging (Olmstead, 2013).

Limitations of this study include the small sample size and difficulty in recruiting patients across two clinic sites. For our sample size, we were underpowered (power ranging from 17% to 56%) to detect smaller differences (10 - 20%) in response rates across various subgroups. Most women that were approached to complete the survey were willing to participate, but often during busy clinics no patients were offered participation. Another limitation is the lack of questions about education, income levels and insurance status, which are associated with more health care knowledge. In the future we would also collect additional detailed information on obstetrical histories, miscarriages, and actual infertility therapy. Because the questions were anonymous, no clinical data could be linked to responses. We are unable to include causes of difficulty conceiving, the number of recent or previous visits to the obstetrics or fertility clinic, or any current or past treatment in the analysis. In the future it would be ideal to collect an equal and larger number of surveys from both sites to better asses how results compared across these groups. Future studies might also involve a baseline survey followed by a short web based educational video intervention and post intervention quiz. Previous studies from Canada and Australia have both supported and questioned the benefit of online education to increase fertility awareness and support informed family decision making (Daniluk & Koert, 2015; Wojcieszek & Thompson, 2013).

In summary, we found that a diverse group of women at our institution had more awareness of the effects of reproductive aging on pregnancy risks compared to age-related fertility decline. Our study demonstrated a unique finding of African America women having less awareness compared to other ethnic groups, of factors specifically affecting pregnancy over 35 years of age. Surprisingly, women that had been pregnant before or received fertility therapy, in whom we expected would be more aware, also did not report high levels of knowledge of age-related fertility decline. Our findings are particularly concerning given the strong reproductive health guidelines from women's health societies, as well as the fact that more women are delaying childbearing. Women continue to need more education and better reproductive counseling to allow them to make the best reproductive life plans. Public education for fertility and reproductive planning is best done, in patient's opinion; through health care providers or educational websites (Lundsberg *et al.*, 2014). This type of directed nonjudgmental counseling will allow women to better plan their reproductive lives. We need to help dispel the reproductive myths and continue to follow the ACOG and the ASRM women's educational guidelines to discuss age-related fertility decline at visits with women of reproductive age.
